# Dynamical heart beat correlations during running

**DOI:** 10.1038/s41598-020-70358-7

**Published:** 2020-08-12

**Authors:** Matti Molkkari, Giorgio Angelotti, Thorsten Emig, Esa Räsänen

**Affiliations:** 1 Computational Physics Laboratory, Tampere University, 33720 Tampere, Finland; 2 Laboratoire de Physique Théorique et Modèles Statistiques, CNRS UMR 8626, Université Paris-Sud, Université Paris-Saclay, 91405 Orsay Cedex, France; 3 Joint MIT-CNRS Laboratory (UMI 3466), MultiScale Materials Science for Energy and Environment, Massachusetts Institute of Technology, Cambridge, MA 02139 USA

**Keywords:** Power law, Statistical methods, Physiology, Applied mathematics, Scientific data, Nonlinear phenomena

## Abstract

Fluctuations of the human heart beat constitute a complex system that has been studied mostly under resting conditions using conventional time series analysis methods. During physical exercise, the variability of the fluctuations is reduced, and the time series of beat-to-beat RR intervals (RRIs) become highly non-stationary. Here we develop a dynamical approach to analyze the time evolution of RRI correlations in running across various training and racing events under real-world conditions. In particular, we introduce dynamical detrended fluctuation analysis and dynamical partial autocorrelation functions, which are able to detect real-time changes in the scaling and correlations of the RRIs as functions of the scale and the lag. We relate these changes to the exercise intensity quantified by the heart rate (HR). Beyond subject-specific HR thresholds the RRIs show multiscale anticorrelations with both universal and individual scale-dependent structure that is potentially affected by the stride frequency. These preliminary results are encouraging for future applications of the dynamical statistical analysis in exercise physiology and cardiology, and the presented methodology is also applicable across various disciplines.

## Introduction

The increasing popularity and accuracy of wearable devices and sensors present new opportunities to study human physiology in a continuous, non-invasive manner for a huge number of subjects under real-world conditions. These devices enable the measurement of a plethora of physiological and mechanical signals such as the heart rate, beat-to-beat (RR) intervals, overall motion via GPS, motion of specific body locations via accelerations, and skin temperature. These data can be recorded in real time, often at 1 s intervals, and uploaded to web services. To date, most recorded data are not analyzed in scientific rigour due to a lack of suitable models for the dynamics of physiological signals under various intensities of exercise load, and also due to restricted availability of the data (property of industry and users). This limits opportunities for a better understanding of complex physiological processes, diagnostics and monitoring for patients in rehabilitation, and the optimal training of athletes.

However, it has been long known that a variety of physiological conditions and cardiac diseases affect heart rate variability (HRV) and the correlations in RR intervals^1^. In exercise physiology, HRV is often used at rest to evaluate recovery, fatigue and overtraining. It is known that during exercise the overall variability of the RR intervals (RRI) is strongly suppressed. Regardless, the RRI correlations contain valuable information even during exercise^2–4^. For example, the possibility to determine certain physiological thresholds, such as the anaerobic threshold, from the frequency spectrum of HRV has been examined^5,6^. Often the relative importance of low-frequency (LF: 0.04–0.15 Hz) and high-frequency (HF: 0.15–0.4 Hz) spectral power is studied during exercise. Using this concept as a measure of the relative sympathetic (SNS) and parasympathetic nervous system (PNS) activity, it has been shown that the PNS activity decreases dramatically during exercise^7^. In contrast, the SNS activity remains unchanged past the first ventilatory threshold before increasing abruptly^7^. However, the use of the HF/LF ratio to measure cardiac sympatho-vagal balance has been criticized^8^. Moreover, it is known that Fourier decomposition of dynamic signals is often hampered by non-stationarity.

To overcome the complications of Fourier methods and non-stationarities, we base our analysis on detrended fluctuation analysis^9^ (DFA), which was developed to measure correlations in non-stationary time series by utilizing systematic detrending^9–11^. Furthermore, we are interested in analyzing real world exercises recorded with readily available commercial sports watches. Hence, we study real-time correlations of RRIs during marathon races (group M) and freeform training runs (group T). Such uncontrolled data may be plagued by severe non-stationary conditions, and the conventional division into short- and long-scale DFA exponents^10,12^ is likely to be insufficient. To this end, we introduce dynamic DFA (DDFA) for the accurate determination of time- and scale-dependent scaling exponents $\alpha (t,s)$ with high temporal resolution. To check the consistency of our methodology, we also apply similar dynamic approach to partial autocorrelation functions (PACFs) to obtain their dynamic counterpart (DPACF).

## Results

Our main result is the discovery of scale-dependent anticorrelations ($\alpha <0.5$) in the RRIs during running that vary with the heart rate. The anticorrelations appear after the HR exceeds a subject-specific threshold. Their magnitude and the scale with the most dominant anticorrelations changes with exercise intensity. We find that the DDFA method can reliably determine the *dynamic, scale-dependent* scaling exponent $\alpha (t,s)$ (please see the Supplementary Information for its numerical validation). Hence, it provides a powerful method for measuring multiscale correlations of non-stationary physiological signals. The results from the DDFA and DPACF methods are found to be mutually consistent.

### Marathon races

**Figure 1:**
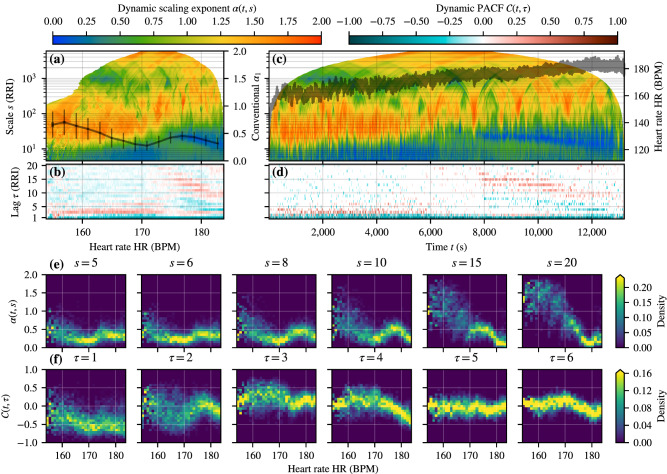
Beat-to-beat (RR) interval correlations for the Marathon race of subject M1. Note that the upper-left and upper-right color bars refer to (**a**, **c**) and (**b**, **d**), respectively. (**a**) Color-coded dynamic (DDFA-1) scaling exponent $\alpha (t,s)$ on different scales *s* (y-axis) as a function of binned HR (x-axis). Here $\alpha (t,s)$ is averaged over those dynamic segments, whose average HR falls within 0.1 BPM wide bins. The values for empty bins are linearly interpolated if the gap does not exceed 0.5 BPM. The black solid line shows the mean together with the standard deviation (thin lines) and the the standard error of the mean (thick lines, barely visible) of the conventional short-scale (4–16 RRIs) scaling exponent $\alpha _1$. The exponent is computed in moving windows of 50 RRIs in HR bins of 2 BPM. (**b**) Color-coded partial autocorrelation functions (DPACF-0) $\mathcal {C}(t,\tau )$ with different lags $\tau$ (y-axis) as a function of the binned HR. (**c**) Similar to (**a**) but as a function of time during the marathon race. The instantaneous heart rate is overlaid on the data. (**d**) Similar to (**b**) but as a function of time. The values that do not pass the non-zero significance test as described in the text are shown in white. (**e**) Probability density histogram for $\alpha (t,s)$ for different scales *s* as a function of the HR. (**f**) Probability density histogram of the DPACF-0 for different lags $\tau$ as a function of the HR. The histograms in (**e**, **f**) consist of 31-by-31 bins, and the probability densities are separately normalized for each HR bin, so that they better depict the distributions as a function of the HR instead of measuring the prevalence of different HR regions. Furthermore, the color bar is capped at the 99.5th percentile to avoid outliers dominating the color scale.

Figure 1 demonstrates our methods applied to a single marathon run (subject M1) of group M. The color-coded value of the scale-dependent exponent $\alpha (s)$ is shown in the first row as a function of the binned heart rate (HR) (Fig. 1a) and also as a function of running time *t* (Fig. 1c). Over the studied scales *s* from 5 to 5000 heart beats, the scaling exponent $\alpha (s)$ exhibits complex behavior that could not be adequately described by the conventional division into short- and long-range scaling exponents. We consider the HR-dependent shift to anticorrelated RRIs at the shortest scales $s \lesssim$ 10–30 as the most interesting of our observations. As the heart rate increases the anticorrelations extend to slightly longer scales until there is a qualitative change at approximately 175 BPM. The strongest anticorrelations shift from the shortest scales to roughly 20 beats, and gradually refill the shortest scales as the HR is increased even further. At larger scales $s \gtrsim 100$ the RRIs become mostly non-stationary ($\alpha > 1$, fractional-Brownian-motion-like behavior). In contrast, a typical 24-h RR-tachogram of a healthy subject at rest usually displays 1/*f* or pink noise on long time scales (or low frequencies $\lesssim 0.05$ Hz), corresponding to $\alpha =1$, and larger values for $\alpha$ at the shortest scales or higher frequencies^1^.

The black curve in Fig. 1a corresponds to the conventional DFA exponent $\alpha _1$ over the scales from 4 to 16 heart beats. It shows almost linear decrease to values around 1/2 and below, when the DDFA exponent $\alpha (s)$ displays short-scale anticorrelated behavior. However, this simpler estimate is not sufficient for uniquely distinguishing the presence of anticorrelations from their shift to slightly longer scales. To explore the scale dependence of the anticorrelations in more detail, we show in Fig. 1e the probability density for the values of $\alpha (s)$ for six different scales *s* from 5 to 20 heart beats as a function of the binned HR. On all six scales, the probability is maximum for $\alpha <1/2$ with a HR dependent modulation and an absence of anticorrelations on the two largest scales $s=15, 20$ for lower beat rates.

In order to estimate the relevant time scales of the physiological processes behind the observed anticorrelated beat intervals, we have also performed a DPACF analysis. The result is shown for lags between 1 and 20 heart beats in Fig. 1b and d. The PACF reveals direct anticorrelations (negative values) after a time lag of 1 and 2 beats, starting at low exercise intensities, and additional anticorrelations up to about 10 beats beyond HR of about 175 BPM, being consistent with the DDFA results. The probability density of the DPACF values for lags between 1 and 6 beats, shown in Fig. 1f, confirm dominant direct anticorrelations on the shortest time scales of 1–2 beats, and 4 beats for high exercise intensities (here HR $\gtrsim 170$).

Subject M1 as the chosen example has the most prominent anticorrelations and particularly simple, almost linear, trend in the HR over the whole marathon. The *individual* DDFA and DPACF results, similarly to Fig. 1, for all the subjects of group M are shown in Supplementary Fig. S5. The results share qualitative similarities across the subjects, as they all exhibit short-scale suppression of correlations and the appearance of anticorrelations as a function of the HR. However, some differences are also apparent, as only three subjects (M1, M3, and M7) show the shift of the anticorrelations to elevated scales at the highest exercise intensities. Some short-scale anticorrelations, particularly for subject M6 and to some extent for M4 and M5, also appear at elevated scales, but these happen at lower intensities and are likely different in origin. Regardless, additional research is required to determine the effect of individual strains relative to standard physiological thresholds on the results.

**Figure 2:**
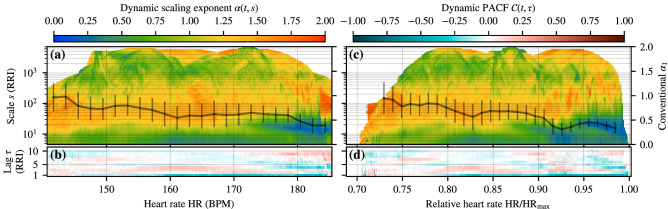
Aggregate beat-to-beat (RR) interval correlations as a function of heart rate for *all* subjects of group M. (**a**) Average values for $\alpha (t,s)$ for each scale *s* (y-axis) and HR bin (x-axis). The solid line depicts the conventional short-range $\alpha _1$. (**b**) Average values for $\mathcal {C}(t,\tau )$ for each lag $\tau$ (y-axis) and HR bin (x-axis). In (**a**, **b**), the data is processed as in Fig. 1. (**c**, **d**) Similar to (**a**, **b**) but as a function of the relative HR. In (**c**, **d**), the data is processed as in Fig. 1, but with the distinction that the relative HR bin width is 0.001, the interpolation threshold is 0.005, and the bin width for the conventional short-scale exponent $\alpha _1$ in (**c**) is 0.01.

To further study the consistency of the results between the different subjects, the aggregated DDFA (top) and DPACF (bottom) results for *all members of group M* are shown in Fig. 2 as a function of both the absolute (left) and relative (right) HR. The most notable features are the high-intensity elevated-scale anticorrelations starting to appear at 87% and 95% relative HR, or at the absolute HR of 175 BPM (this congruence on the absolute scale is likely to be coincidental). In these ranges also the conventional DFA exponent $\alpha _1$ (black curve) drops slightly below 1/2, but its limitations are apparent, as it is based on linear regression over the scales of 4–16 beats. The anticorrelations at lower intensities (approximately 155–175 BPM) appear to be more condensed on the relative scale (roughly 78–87%, with a more concentrated maximum between 80–85%), which is apparent both on the DDFA results and in the more pronounced dip of the short-scale exponent $\alpha _1$. There is also a band of short-scale suppressed correlations with a trend towards longer scales at even lower relative HR ($\approx$ 72–79%) that is practically indistinguishable on the absolute scale. On the relative scale, the DPACF results also show a sharper transition into anticorrelated behavior at approximately 80% HR for lag $\tau =1$, whereas on the absolute scale the transition is more gradual. These lag $\tau =1$ anticorrelations appear consistently for all the subjects and become stronger with increasing HR, and also appear at longer lags at the regions where the DDFA anticorrelations shift to larger scales. Naturally, the aggregated results should be interpreted with care as they represent an average result over all the samples. Secondly, there is uncertainty in the maximum HR values of the subjects. Nevertheless, the results suggest that neither the absolute nor the relative scale is universal for different individuals.

### Freeform training runs

**Figure 3:**
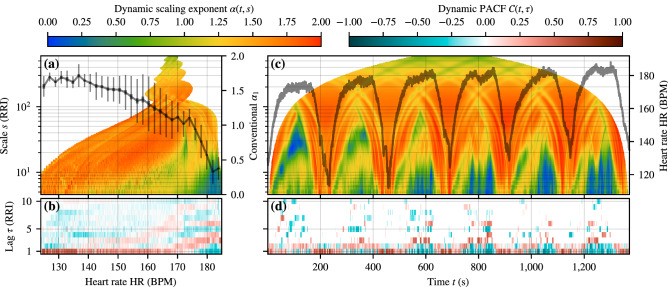
Beat-to-beat RR interval correlations for *one* interval exercise from group T. (**a**) Dynamic scaling exponents (DDFA-1) $\alpha (t,s)$ (colors) and the conventional short-range $\alpha _1$ (solid line) as a function of the binned HR. (**b**) DPACF-0 correlations $\mathcal {C}(t,\tau )$ as a function of the binned HR. (**c**, **d**) As in (**a**, **b**) but as a function of time, and in (**c**) the HR value is overlaid on the data. For details on the data processing, see the caption of Fig. 1.

In order to study the correlations of RRIs over a wide range of exercise durations and intensities, we perform the same analysis for subjects in group T. It is instructive to consider first a single exercise of one subject which is shown in Fig. 3. It consists of six intervals of high-intensity running, each interval lasting about 160 s with the subsequent intervals reaching higher and higher intensities. As a function of exercise time *t* the DDFA exponent $\alpha (s)$ (Fig. 3c) and the PACF (Fig. 3d) consistently reveal strong anticorrelations of RR intervals that develop rapidly after the start of the intense interval. The shortest-scale anticorrelations span to longer and longer scales with increasing HR in the latter intervals. The earlier lower-intensity intervals exhibit anticorrelations at elevated scales, separated by a band of correlations from the shortest scale anticorrelations. This behavior was already suggested by some of the marathon data (M4, M5, and M6), and in a following analysis we will relate these to a distinct band of anticorrelations appearing at moderate exercise intensity. At rest between the intervals the anticorrelations rapidly vanish. The DPACF shows strong lag $\tau =1,2$ anticorrelations, whose magnitudes are in accordance with the short-scale DDFA anticorrelations as observed in group M. The existence of patches of anticorrelations over time lags up to 10 beats is also consistently observed with the elevated-scale DDFA anticorrelations. As a function of HR, the anticorrelated behavior develops rapidly after an intensity threshold ($\approx$ 175 BPM) (see Fig. 3a). The elevated-scale anticorrelations are visible as a spike of suppressed correlations at approximately 172  BPM with a weak tail towards short scales and lower intensities. This latter phenomenon is more visible in the DPACF data (Fig. 3b) as a weak band of anticorrelations surrounded by correlated bands at shorter and longer lags.

**Figure 4:**
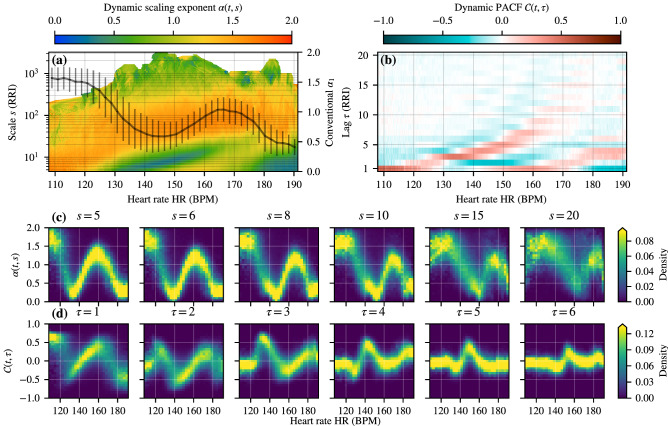
Aggregate beat-to-beat (RR) interval correlations for *all* the exercises of one subject (T07) in group T. (**a**) Average values for $\alpha (t,s)$ for each scale *s* (y-axis) and binned HR (x-axis). The solid line shows the conventional short-range $\alpha _1$. (**b**) Average values for $\mathcal {C}(t,\tau )$ for each lag $\tau$ (y-axis) and binned HR (x-axis). (**c**) Probability density histogram for $\alpha (t,s)$ for different scales *s* as a function of the HR. (**d**) Probability density histogram for $\mathcal {C}(t,\tau )$ for different lags $\tau$ as a function of the HR. Note that the probability densities are separately normalized for each HR bin. For details on data processing, please see the caption of Fig. 1.

Next, we study the typical behavior of RRI correlations when averaged over many running exercises of different intensity and duration, but for the same subject to avoid effects due to individual variability. The corresponding aggregated results from DDFA and DPACF analysis for the subject T07 are shown in Fig. 4, representing a total running distance of 1889 km. For this large data set, we obtain good statistics for the aggregated data and expect them to provide a reliable representation of the typical RRI correlations as function of the exercise intensity. Indeed, both DDFA exponent $\alpha (s)$ and DPACF clearly show two distinct bands of anticorrelated RRIs (see Fig. 4a,b). The first band at moderate exercise intensity ($\approx$ 125–170 BPM) displays a distinct, approximately exponential, trend in the DDFA anticorrelations shifting to longer scales as a function of the HR. It is plausible that the elevated scale anticorrelations appearing at lower intensities in Fig. 3 and for M4, M5, and M6 originate from this band of anticorrelations. The corresponding band in the DPACF results is split by a band of strong positive correlations. The latter band of anticorrelations at high exercise intensities ($\gtrsim 175$ BPM) does not show a clear trend as a function of the HR, although there is tendency towards spanning to longer scales with increasing intensity. Notably, the anticorrelations remain present even at the shortest scales and lag. This is in contrast to some of the marathon data, where the highest-intensity anticorrelations appear at elevated scales. This could be due to the different nature of the exercises, as in the marathon races these anticorrelations appear after prolonged exercise at high intensity, and changes in, e.g., body temperature or electrolyte balance may influence the results. On the other hand, in the discussion section we make an argument that this could be due to interactions with the stride frequency. The conventional $\alpha _1$ indicates the suppression of correlations that is consistent with the DDFA anticorrelations when taking into account its limitation to the scales of 4–16 beats. The $\alpha _1$ is clearly insufficient to capture anticorrelated behavior concentrated on thin bands of scales. Figure 4c,d shows the probability density plots of $\alpha (s)$ and PACF values for different scales *s* and lags $\tau$, respectively. The existence of two regions with anticorrelated RRIs is clearly visible. They are separated by a region with positive correlations (or $\alpha >1/2$).

The aggregated data for all the subjects of group T are shown in Supplementary Fig. S4. Most subjects show common qualitative similarities in the form of two anticorrelated bands as described for T07. However, for some subjects the split into the two anticorrelated regions is not that clear; particularly there is the lack of correlated shortest scale behavior separating these two regions. In the absence of the correlated bands the behavior is remarkably simple; the higher the intensity, the more prominent the anticorrelations are in both magnitude and scales covered. In addition to individual intrinsic cardiac variability, a possible explanation could be different training practices and external conditions, as for example T05 shows behavior that is most similar to the marathon data. Another explanation could be highly regular running motion, which could promote correlations induced by, e.g., muscle contractions and blood pressure variations, which is an argument set forth in the next section. It is also worth noting that T11 reported problems with the chest strap, and as a result his data has unusually high amount of missed beats (up to 50%). Despite of this, two regions of suppressed correlations are present that are consistent with the other subjects, highlighting the robustness of the methodology.

We assessed the suitability of higher order detrending for our analysis and decided to employ DDFA-1 due to the following reasons: (1) the qualitative behavior remains the same at the shortest scales, which is the most interesting region for dynamic exercise intensity analysis, (2) the short-scale bias in DFA is larger and crossover scales are shifted with higher order methods, (3) higher orders of DDFA appear to require longer dynamic segments for similar statistical accuracy and have increased computational cost.

Finally, we point out that it is important to check the reliability of our DDFA and DPACF methods with respect to trends. Hence, we have filtered the data of subject T07 according to the condition that the standard deviation of the HR within the dynamic segments is smaller than the values for certain quantiles. We find that the observation of the bands with anticorrelations is robust and independent of the choice of the quantile filter. In fact, the anticorrelations appear stronger when limiting to dynamic segments with less HR variation, as the averaging is not performed over segments with transient changes in the HR that could lead to spurious correlations. The exact results of this analysis for six different choices of quantiles are shown in Supplementary Fig. S6.

## Discussion

It is important to understand the physiological mechanism causing the observed anticorrelations. Due to the lack of time series for other physiological variables, we can present below only simple arguments that we consider to be potentially relevant for explaining the observed dynamic correlations. First, we point out that there are three physiologically relevant time scales that fall into the range over which the anticorrelations occur: (1) the stride frequency which is typically around 85 strides per leg and per minute^13^, (2) the respiration cycle which is typically three to five heart beats long, and (3) arterial blood pressure fluctuations, i.e., the so-called Mayer waves, which result from an oscillation of sympathetic vasomotor tone and is of the order of 10 s^14^.

All three processes are cyclic and hence can induce periodic modulations to the heart rate through hemodynamics. Such periodicities could result in anticorrelated RRIs when observed at scales similar to the period measured in heart beats. Furthermore, the overall heart rate variability is reduced under exercise due to withdrawal of cardiac vagal tone and parasympathetic control^2–4^, i.e., the local short-term RRIs are more regular without 1/*f*- or Brownian-like diffusion for extended periods of time. Therefore, subtler patterns should become more discernible, as they are not masked by the complex fluctuations of a healthy heart under resting conditions. Similarly, the relative magnitudes of the modulating signals could affect the scale-dependence of the anticorrelations. If some of the effects is much stronger, it will mask higher frequency periodicities as they will appear correlated when superimposed on the stronger lower frequency oscillations. Additionally, when a periodic signal is sampled at discrete intervals, the result is a new signal whose period depends on the sampling frequency. This effect is manifested, e.g., when the influence of the blood pressure variations due to the stride frequency is sampled at each heart beat.

This latter phenomenon could explain some of the qualitative differences in the dynamic correlations between the subjects. For some subjects (particularly T08, but also T02, T03, T10, and T12) the RRIs show clearly defined behavior under exercise, becoming short-scale anticorrelated at moderate intensity, with the magnitude and the scale of the anticorrelations increasing in conjunction with intensity. In contrast other subjects exhibit more complex RRI-correlations where the simple anticorrelations are interrupted by bands of decreased or altered correlations at shorter scales (please see Supplementary Figs. S4 and S5 for the individual RRI-correlation plots as a function of the HR). These more correlated bands appear at heart rates corresponding to sampling frequencies where typical stride frequencies would look correlated at the shortest scales. If these bands arise from the stride frequency, that could also explain the better congruence on the absolute HR scale in Fig. 2, as there is generally less variance in the stride frequencies than in the maximum heart rates.

These considerations would imply that the (anti)correlations arise from underlying universal cardiolocomotor mechanisms, but detailed response to exercise may depend on individual physiology, biomechanics of running and training status^6^. Furthermore, the onset of the anticorrelations, their strength, and scales of appearance show individual variability. Studying the relationship of these variables to standardized thresholds and markers in exercise physiology could allow utilizing the dynamic correlations for monitoring the exercise intensity in real-time without the knowledge of parameters such as the maximal oxygen uptake (VO2max) or maximum heart rate. We are aware of the possibility that the universal emergence of anticorrelations at elevated heart rates is most likely affected by other physiological factors beyond the ones discussed here. Clearly, further research is required, but the approach herein provides a promising avenue forward.

## Conclusions

Our main result is the discovery of multiscale anticorrelations in RR intervals during running exercises under real-world conditions. The anticorrelations have a dynamical structure that depends on the exercise intensity as measured by the heart rate. The characteristics of the dynamical structure are revealed by our methodology, in particular the dynamic detrended fluctuation analysis and dynamic partial autocorrelation functions, which we anticipate becoming useful tools in data analysis across various disciplines. While we have demonstrated the capability to study the dynamical RRI correlations during varying real-world circumstances, a more systematic evaluation of the methodology is required to control for exercise conditions.

The observed anticorrelations appear on short scales (a few beats) at low to moderate exercise intensities. As the intensity is increased, the anticorrelations increase in magnitude and span to longer scales (up to 20–30 beats). This simplified picture is complicated by correlations arising potentially from interactions with the regular running motion when the stride frequency is appropriately proportional to the heart beat. These correlations mask the anticorrelated behavior on bands of increasing or decreasing scales at moderate and high exercise intensities, respectively. At rest, e.g., between running intervals, the anticorrelations rapidly vanish, and appear immediately when the intensity is increased again. These changes happen before the HR saturates at the level necessary to maintain the ongoing exercise intensity. Hence, our findings allude the possibility of quantifying the relative exercise intensity by measuring the dynamic correlation exponent $\alpha (t,s)$ in real time during exercise.

This report of our initial findings serves as a prelude for highlighting the potential of the dynamic correlation analysis so that further advances could be pursued. It is highly desirable to develop a theoretical model for the complex dynamics of the cardiovascular feedback loops during high-intensity exercise load that can explain the observed time scales for the anticorrelated RR intervals. Clearly, a more systematic study with subjects performing specific exercise protocol should be performed to verify our observations. Besides, a thorough validation and calibration of our results with data collected during running exercise in a physiology laboratory is a natural next step for our study to relate the changes in the dynamic correlations to standard exercise physiology models. The inclusion of accelerometer data, from which the stride frequencies could be derived, would facilitate the verification of the running modalities as a possible cause for the bands of correlations present for some subjects.

Such controlled and systematic studies are not only necessary to elucidate the speculative nature of the results herein, but they are further motivated by potentially enabling the application of this methodology in exercise physiology. We expect that the reported RR interval correlations are suitable to represent a dynamical “fingerprint” of the exercise-induced cardiovascular load. Hence, our methodology—which could be integrated with the present devices on the market—has a potential to become a new tool in real-time exercise monitoring without previous knowledge of maximal thresholds such as the maximum hearth rate and lactate or ventilatory thresholds.

## Materials and methods

### Heart rate data during exercise

We study real-time correlations of RRIs during exercises of various intensities. All heart rate data for this study have been collected during regular running training and racing under real-world conditions, i.e., outside the laboratory. Two groups of data were used for our theoretical analysis. The first group of data was recorded by human volunteers during their regular running training with freely chosen intensity and volume (group T). The study period was at least 4 weeks, and some subjects provided data over a longer period of time. We obtained institutional approval and informed consent (Massachusetts Institute of Technology Committee on the Use of Humans as Experimental Subjects exemption for the employed protocol has been granted under protocol no. 1711132002). The research was performed in accordance with the rules and regulations set by the participating universities. This group involved 12 volunteers (5 female, 7 male, with an age span from 27 to 65 years). Their performances span a wide range from top national level to recreational runners: the personal bests in 10 km range from 29 min 31 s to 44 min 57 s, in marathon from 2 h 43 min 20 s to 4 h 26 min 3 s.

During exercises, heart rate (HR), RR intervals (RRI), running velocity and distance were recorded using a Garmin heart rate monitor HRM4-Run and a GPS watch (Forerunner 935, Garmin Inc., Olathe, KS, USA). A previous study has investigated and validated the accuracy of this HRM^15^. The data were recorded by the GPS watch in the Flexible and Interoperable Data Transfer (FIT) format^16^ and subsequently uploaded by the subjects to a web service that we had launched for this study. The total number of exercise files analyzed per subject (samples) varied between 18 and 261, with total covered distances from 150 to 1889 km.

The second group of data was obtained by selecting randomly the marathon races of 7 subjects from data uploaded to the Polar Flow web service^17^ (group M). Within registration to Polar Flow, the subjects have given their consent for the use of their data for research purposes. The metadata were provided by the users of this web service (all male, with an age span from 28 to 53 years, and marathon finishing times between 3 h 30 min and 4 h 17 min). HR and RRIs were recorded for this group of subjects with a Polar heart rate monitor H10 and a Pro Strap (Polar Electro Oy, Kempele, Finland). Recently, the RR signal quality of this HRM has been shown to be excellent from low- to high-intensity activities in comparison to a ECG Holter device^18^. In both groups T and M, the subjects provided their maximum and resting heart rates. Summaries of all the metadata for the two groups are shown in Supplementary Table S1.

As ECG data is not available, we do not attempt to remove ectopic beats or other artifacts based on physiological criteria. Therefore we merely remove technical artifacts, such as missed beats, that can be isolated with reasonable certainty. The details for this data preprocessing are provided in Supplementary Appendix A.

### Conventional methods

For comparison we apply ordinary detrended fluctuation analysis^9,11^ to the RRI time series. By computing the root-mean-squared fluctuations *F*(*s*) around local trends at multiple scales *s*, the method assesses power law scaling relations $F(s) \propto s^{\alpha }$ characterized by the scaling exponent $\alpha$. In the context of HRV, typically two exponents are determined, for short- ($\alpha _1$) and long-scale ($\alpha _2$) correlations, respectively^10,12^. We extract the conventional short-scale (4–16 RRIs) scaling exponents $\alpha _1$^10^ in segments consisting of 50 RRIs. We compute the fluctuation functions in maximally overlapping windows for enhanced statistical properties^19^. A summary of the DFA method is provided in Supplementary Appendix B. We also provide a helpful summary of partial autocorrelation functions in Supplementary Appendix C before introducing their dynamic counterparts here.

### Dynamic segmentation

The dynamic behavior of the time series can be studied by performing the analysis in moving temporal segments. However, to guarantee sufficient statistical accuracy, the length of these segments is dictated by the largest scale *s* (DFA) or the lag $\tau$ (PACF), resulting in diminished temporal resolution for small scales. Therefore, we propose a dynamic segmentation procedure, where the segment length is varied as a function of the scale or the lag:

1. Choose a function for determining the segment lengths $\ell (s)$ as a function of the scale *s*. Here we adopt a simple linear relationship $\ell (s) = as$ where $a$ is a constant. Smaller values increase the temporal resolution but also the statistical noise. The dynamic length factor $a$ itself may also be varied for different scales.
2. For each scale divide the time series into segments of length $\ell (s)$. The segments themselves may be overlapping if desired for smoother results. Identify the segments $\mathcal {S}_{s,t}$ by their temporal indices *t*, which may be, e.g., the mean time within the segment or any other suitable quantity.

#### Dynamic detrended fluctuation analysis (DDFA)

The dynamic segmentation together with the maximally overlapping windows in the DFA scheme enables the following procedure for dynamic DFA (DDFA):

1. Perform the dynamic segmentation for each scale *s*. The value of $a=5$ was found to be an acceptable value for the dynamic length factor, which is employed in all of our DDFA calculations.
2. Utilizing overlapping windows, compute the fluctuation function in each segment $\mathcal {S}_{s,t}$ at scales $\{s-1, s, s+1\}$. Denote the logarithmic fluctuation function at these scales by $\tilde{F}_{t}(s-1), \tilde{F}_t(s)$ and $\tilde{F}_{t}(s+1)$, respectively.
3. In each segment, compute the dynamic scaling exponent $\alpha (t,s)$ by the finite difference approximation^20^
  1 $$\begin{aligned} \alpha (t,s)&\approx \big [ h_{-}^2\tilde{F}_{t}(s+1)+ \left( h_{+}^2-h_{-}^2\right) \tilde{F}_t(s)- h_{+}^2\tilde{F}_{t}(s-1)\big ] / \big [ h_{-}h_{+}\left( h_{+}+ h_{-}\right) \big ]\text {,} \end{aligned}$$
  where $h_{-}= \log (s) - {\log (s - 1)}$ and $h_{+}= {\log (s + 1)} - \log (s)$ are the logarithmic backward and forward differences. Fluctuation functions computed with maximally overlapping windows are empirically found to be smooth enough to permit the direct application of the finite difference scheme.

The performance of the method is numerically validated by applying it to simulated time series with known properties. Supplementary Appendix D explains the details for analytically obtaining the theoretically expected scale-dependency of DFA scaling exponents for different processes. In Supplementary Appendix E these theoretical results are utilized for confirming the acceptable performance of the DDFA method.

#### Dynamic partial autocorrelation function (DPACF)

In order to obtain a *local* estimate of the partial autocorrelation function $\mathcal{C}(\tau )$ we compute it using an approach similar to that of the DDFA algorithm. The steps of this approach can be summarized as follows:

1. Perform dynamic segmentation for each lag $\tau$. The value of $a=10$ was found to be an acceptable value for the dynamic length factor, which is utilized in all of our DPACF calculations.
2. In each segment $\mathcal {S}_{\tau ,t}$, perform polynomial detrending of order *m*.
3. For each segment, compute $\mathcal{C}(\tau )$ by, for example, solving the Yule-Walkers equations with the Levinson-Durbin recursive scheme^21^. Choose each time for the maximum lag the parameter for which we are estimating the partial autocorrelation function. Denote this dynamic PACF by $\mathcal{C}(t,\tau )$.

Resorting to the central limit theorem, it is a known result that the partial autocorrelation function is approximately non-zero at 5% significance level if ${\left| \mathcal{C}(t,\tau )\right| }< 1.96/\sqrt{\ell (\tau )}$. The evaluation of this significance band is statistically valid only if $\ell (\tau )\gtrsim 30$ and therefore if $\tau >3$ for $a=10$.

Notice that in DPACF the detrending is applied to the original time series in contrast to the integrated series in DDFA. Therefore, the results would be expected to be qualitatively similar when the DPACF detrending order is one smaller than the DDFA detrending order *n*. This explanation is complicated by, e.g., the removal of linear correlations in PACF. However, the relationship ${m \approx n - 1}$ is supported by empirical observations.

While both DDFA and DPACF measure dynamic correlations, it is important to realize the qualitative difference between them. DDFA describes the *collective* behavior of all beats over the scale *s*, whereas DPACF considers the average behavior of *individual* beats separated by the lag $\tau$ (with the linear dependence from the preceding lags removed)

## Supplementary information

Supplementary information.

## Data Availability

All data generated and analyzed during the current study are available from the corresponding author upon reasonable request.
