# Supplementary material for: Dynamical heart beat correlations during running
Source: Sci Rep. 2020 Aug 12;10:13627. doi: 10.1038/s41598-020-70358-7 (PMC7423621; doi:10.1038/s41598-020-70358-7)
Supplement: Supplementary file 1 — Supplementary information. [file 41598_2020_70358_MOESM1_ESM.pdf]

Supplementary Information for “Dynamical Heart Beat Correlations during Running”

Matti Molkkari[1,*], Giorgio Angelotti[2], Thorsten Emig[2,3], and Esa Räsänen[1,2]

[1]Computational Physics Laboratory, Tampere University, FI-33720 Tampere, Finland
[2]Laboratoire de Physique Théorique et Modèles Statistiques, CNRS UMR 8626, Université Paris-Sud, Université Paris-Saclay, 91405 Orsay cedex, France
[3]Massachusetts Institute of Technology, MultiScale Materials Science for Energy and Environment, Joint MIT-CNRS Laboratory (UMI 3466), Cambridge, Massachusetts 02139, USA
[*]matti.molkkari@tuni.fi

A Dataset details and preprocessing

We study two datasets of running exercises and races under real-world conditions. These consist of voluntary running exercises at freely chosen intensities and schedules (group T) and marathon races (group M). Metadata for these datasets are presented in Supplementary Table S1.

Artifacts in the data are removed prior to the analysis by utilizing the following scheme:

1. RR intervals below or above the threshold values $\mathrm{RR}_{\mathrm{min}}$ and $\mathrm{RR}_{\mathrm{max}}$ are removed.

2. Compute the local median of the RR intervals $\mathrm{RR}_{\mathrm{med}}(t)$ in a moving window of length l_{med}.

3. Remove RR intervals that fall outside the range $(1 \pm c_{\mathrm{med}})\mathrm{RR}_{\mathrm{med}}(t)$, where the threshold c_{med} is a constant.

The values for the filtering parameters are listed in Supplementary Table S2. Acceptable performance of the filter is manually inspected for all the samples in group M and a representative subset of 17 samples from the group T. The filter is particularly adept at removing too long intervals arising from missed beats, which is the most common error in exercise data[1]. As ECG data is not available, we do not attempt to filter the data based on physiological criteria, and merely remove technical artifacts that can be isolated with reasonable certainty.

B Detrended Fluctuation Analysis (DFA)

Ever since its introduction in the study of correlations in DNA sequences[2], DFA for time series has been widely employed across multiple disciplines such as physics[3,4], medicine[5–7], finance[8], and even music[9,10]. The DFA method has been extensively studied[11–19] and it has been expanded to account for effects such as multifractality[20] and cross-correlations[21].

We briefly summarize the conventional DFA algorithm which has been developed to detect correlations in non-stationary time series[2,5]. First, for a time series $X(j)$ of length N a cumulative summation is performed,

$$Y(k) = \sum_{j=1}^{k} \left(X(j) - \langle X \rangle\right) , \tag{1}$$

where the mean $\langle X \rangle$ of the time series is subtracted, but that is not strictly necessary for DFA[11]. Conventionally, the integrated time series of (1) is divided into non-overlapping windows of length s. In each window w, a local trend is determined as the least-squares fit of a low order polynomial $p_{s,w}(k)$ to the data. (The method is denoted by DFA-n if the degree of the detrending polynomial is n[11].) The fluctuations are measured as the variance from the local trend $p_{s,w}(k)$ in each window: $F^2_{s,w} = \frac{1}{s}\sum_{k\in w}\left(Y(k) - p_{s,w}(k)\right)^2$. These squared fluctuations are averaged over the windows to yield the fluctuation function

$$F(s) = \left\langle F^2_{s,w} \right\rangle^{1/2}. \tag{2}$$

Allowing the windows to overlap enhances the statistical properties of this estimate[22]. When this procedure is repeated for different window sizes, or scales s, a power-law increase of the fluctuations with the window size may be observed,

Supplementary Table S1. Summary of the metadata for the two groups of subjects: Training runs of various durations and intensities (T) and marathon races (M). For group T shown are the age, gender, resting heart rate HR_{rest} and maximum heart rate HR_{max} (as reported by the subject), the personal best (PB) time for 10 km and marathon (time in hh:mm:ss) within 3 years before this study, the number of exercise samples analyzed, the average heart rate HR_{avg} of all samples, and the total distance and duration of all samples. For group M all subjects were male, and shown are their age, resting heart rate HR_{rest} and maximum heart rate HR_{max} (as reported by the subject), marathon finishing time, and average heart rate HR_{avg} during the marathon.

Group T

subject	age [y]	gender	HR_{rest}	HR_{max}	PB 10 km	PB Marath.	samples	HR_{avg}	distance [km]	duration [h]
T01	48	m	40	192	00:36:49	02:50:49	186	151	1527	121.5
T02	27	m	40	193	00:29:31	–	54	130	576	43.5
T03	29	f	43	200	00:36:50	02:47:56	20	155	170	13.7
T04	29	f	50	205	00:42:30	–	103	167	1076	92.8
T05	39	f	–	200	00:44:57	03:40:09	15	176	150	11.7
T06	–	m	40	192	00:35:22	02:43:25	26	153	666	49.3
T07	33	m	42	214	00:33:56	02:43:20	261	154	1889	138.7
T08	37	f	43	200	–	04:26:03	20	149	209	22.1
T09	27	m	48	195	00:31:32	–	21	143	199	16.7
T10	37	f	45	179	–	–	18	154	215	18.1
T11	65	m	47	170	00:43:10	03:13:29	26	134	287	28.2
T12	47	m	48	182	–	03:09:49	53	129	1133	95.4

Group M

subject	age [y]	HR_{rest}	HR_{max}	Finishing time [hh:mm:ss]	HR_{avg}
M1	53	56	185	03:40:09	172
M2	50	46	174	03:36:47	149
M3	28	58	198	04:17:04	171
M4	34	50	195	04:12:36	155
M5	43	55	200	03:49:03	162
M6	39	55	194	04:19:36	155
M7	50	50	200	03:30:33	174

Supplementary Table S2. Data filtering parameters.

Group	RR_{min} (ms)	RR_{max} (ms)	l_{med} (beats)	c_{med}
M	250	600	15	0.026
T	250	1000	11	0.03

Supplementary Table S3. Meaning of values of the DFA scaling exponent α. The qualitative interpretation remains the same for even higher exponents that become discernible with higher-order DFA: For each integral interval the lower and upper halves correspond to originally anticorrelated or correlated increments, respectively.

Scaling exponent	Interpretation	Stationarity
$0 < \alpha < 1/2$	anti-correlated	stationary
$\alpha = 1/2$	white noise	
$1/2 < \alpha < 1$	correlated	
$\alpha = 1$	$1/f$ (pink) noise	
$1 < \alpha < 1\,1/2$	anti-correlated increments	non-stationary, stationary increments
$\alpha = 1\,1/2$	Brownian noise	
$1\,1/2 < \alpha < 2$	correlated increments	

i.e., $F(s) \sim s^{\alpha}$. Here α is a scaling exponent that can be considered as a generalization of the Hurst exponent H [1]. See Supplementary Appendix E below for more details.

However, experimental time series rarely exhibit exact scaling over several scales. Many previous studies have focused on finding a robust determination of the scaling regimes[25,26], or on extracting a *spectra* of scaling exponents $\alpha(s)$[27–31]. These methods are based on the notion that the spectra may be defined as the *local slope* of the logarithmic fluctuation function,

$$\alpha(s) = \frac{\mathrm{d}\left[\log F(s)\right]}{\mathrm{d}\left[\log s\right]}. \tag{3}$$

In the context of HRV, these methods generalize and expand the conventional division into short (4–16 beats) and long-range (16–64 beats) scaling exponents. In practice, the behavior may also change over time, either due to external influences, or the process itself may comprise several distinct intrinsic modes. This paper develops a methodology that takes these temporal variations into account in a consistent manner.

Depending on the value of the exponent α, different degrees of correlations of the time series or its increments can be identified. The meaning of the different ranges for α are summarized in Supplementary Table S3.

The scaling exponent α is related to other scaling exponents in time series analysis. Scale invariance is also observed in the Fourier domain as a function of the frequency f with a power spectral density that scales in the low frequency limit as $P(f) \sim f^{-\beta}$. The exponent β is related to the DFA exponent by the scaling relation $\beta = 2\alpha - 1$[14,15]. In exercise physiology, the power spectrum of heart rate time series is a frequently employed tool to quantify the cardiological response to exercise. However, analyses in the frequency domain are potentially plagued by non-stationarity. For stationary signals, the autocorrelation function $\mathscr{C}(\tau) = \langle X(\tau_0)X(\tau_0+\tau)\rangle$ decays for long lags τ with a power law $\sim \tau^{-\gamma}$. Then the scaling relation $\gamma = 2 - 2\alpha$ holds[16]. For more details on the DFA method and its relation to correlation functions, see Supplementary Appendix D.

C Partial Autocorrelation Function (PACF)

It is instructive to supplement DFA analyses by a direct study of correlations at different time scales by computing the autocorrelation function $\mathscr{C}(\tau)$ and the *partial* autocorrelation function $\mathcal{C}(\tau)$ at lag τ. The latter has been successfully used to identify the best autoregressive (AR) process to fit a time series, using the fact that $\mathcal{C}(\tau) = 0$ for all $\tau > p$ for an AR model of order p[32]. The autocorrelation function $\mathscr{C}(\tau)$ is dominated by trends in the data, suggesting apparent correlations. On the contrary, $\mathcal{C}(\tau)$ is less affected by trends due to the subtraction of the linear dependence on intermediate lags from the autocorrelation function. If the time series contains oscillations, $\mathscr{C}(\tau)$ shows a periodic pattern with a frequency that modulates the data. On the contrary, $\mathcal{C}(\tau)$ shows anticorrelations. The lags τ for which $\mathcal{C}(\tau)$ assumes negative values can be related to the periodicity of the oscillations. More specifically, for a time series $X(\tau)$ the partial autocorrelation function is given by

$$\mathcal{C}(\tau) = \left\langle [X(\tau_0) - \hat{X}_{\tau_0\tau}(\tau_0)][X(\tau_0+\tau) - \hat{X}_{\tau_0\tau}(\tau_0+\tau)] \right\rangle \tag{4}$$

[1] Originally it was the exponent in Hurst's R/S analysis[23], and Mandelbrot and van Ness used it as a parameter for defining fGn and fBm[24]. For these processes it can be related to the (asymptotic) DFA scaling exponent (fGn: $\alpha = H$, fBm: $\alpha = H+1$). In the $0 < \alpha < 1$ range the relationship (between R/S and DFA exponents) is approximate, and for fGn/fBm holds asymptotically for large scales. For $\alpha > 1$ the interpretation $\alpha = H+1$ is essentially due to the definition of fBm. It also could be argued to hold, at least approximately, for processes that are defined as the cumulative sum of another process, similarly as the (discrete) fGn/fBm.

where $\hat{X}_{\tau_0\tau}(\tau')$ is the best linear predictor, determined by

$$\hat{X}_{\tau_0\tau}(\tau') = c_0 + \sum_{i=1}^{\tau-1} c_i X(\tau_0 + i)\,, \tag{5}$$

where the coefficients c_i are determined by the conditions

$$c_0 + \sum_{i=1}^{\tau-1} c_i \left\langle X(\tau_0 + i)\right\rangle = \left\langle X(\tau')\right\rangle\,, \tag{6}$$

$$c_0 \left\langle X(j)\right\rangle + \sum_{i=1}^{\tau-1} c_i \left\langle X(\tau_0 + i)X(j)\right\rangle = \left\langle X(\tau')X(j)\right\rangle \tag{7}$$

for $j = \tau_0 + 1, \ldots, \tau_0 + \tau - 1$. The function $\mathcal{C}(\tau)$ can be computed practically from the Yule-Walker equations[32]. The relation between the AR fits and $\mathcal{C}(\tau)$ is useful in the performed analysis. Indeed, it has been shown that a signal with non-trivial periodic behavior can be, for short time scales, successfully fitted by an AR process, and its dominant frequency of oscillation can be extracted from the estimated coefficients. These findings have been also confirmed by DFA[33].

D Additional remarks on DFA for the validation of DDFA

In this section we provide some known theoretical results for the conventional DFA algorithm to support the validation procedure presented in the following section. In DFA, the range of detectable exponents is determined by the degree of detrending, n, and is given by $0 \leq \alpha \leq n+1$[15]. While the existence of values $\alpha > 1$ may be criticized as a failure of the detrending procedure[34], they may also be understood as an advantage of the method for allowing meaningful quantification of non-stationary processes[18,19]. The detrending may be considered successful if it achieves the statistical equivalence over the DFA windows, so that the fluctuation function $F(s)$ does not depend on the window[18,19]. This condition is fulfilled for DFA-n with time series exhibiting polynomial trends of degree $n-1$. In general, for two *uncorrelated* signals $X_A(t)$, $X_B(t)$ (random processes or trends), a superposition principle holds, stating that the squared fluctuation function of the sum $X_{A+B}(t) = X_A(t) + X_B(t)$ is given by $F^2_{A+B}(s) = F^2_A(s) + F^2_B(s)$[12].

For stationary processes and for non-stationary processes with stationary increments the fluctuation function F_s does not depend on the window and may be analytically computed[16,17]. Its squared value is determined as the weighted sum of the autocovariance function $\hat{C}(\tau) = \langle X(\tau_0)X(\tau_0+\tau)\rangle - \langle X\rangle^2$ in the former case, and that of the variogram $S(\tau) = \left\langle [X(\tau_0+\tau) - X(\tau_0)]^2 \right\rangle$ in the latter case,

$$F_s^2 = \sum_{j=-s+1}^{s-1} G(j,s)\hat{C}(j) \tag{8}$$

$$F_s^2 = -\sum_{j=1}^{s-1} G(j,s)S(j) \tag{9}$$

with the weight function $G(j,s)$ given by [2]

$$G(j,s) = \frac{1}{s}\sum_{k=1}^{s-|j|} a_{k,k+|j|}, \tag{10}$$

where $a_{k,k'}$ are the elements of the matrix

$$\mathbf{A} = \mathbf{D}^\top \left[\mathbf{I} - \mathbf{B}^\top \left(\mathbf{B}\mathbf{B}^\top\right)^{-1}\mathbf{B}\right]\mathbf{D}, \tag{11}$$

where the elements $d_{i,j}$ of the matrix $\mathbf{D}$ are unity if $i \geq j$ and zero otherwise[16,17]. The effect of detrending is incorporated into the so-called design matrix $\mathbf{B}$ of least squares regression, which for DFA-1 is given by[17]

$$\mathbf{B} = \begin{bmatrix} 1 & 1 & \cdots & 1 \\ 1 & 2 & \cdots & s \end{bmatrix}. \tag{12}$$

[2] We adapt the notation from Ref.[17] with the following changes: The factor s^{-1} is included in the weight function $G(j,s)$ that is symmetrically extended for negative j by $G(-j,s) = G(j,s)$. This allows for a more succinct notation for Eqs. 8 and 9.

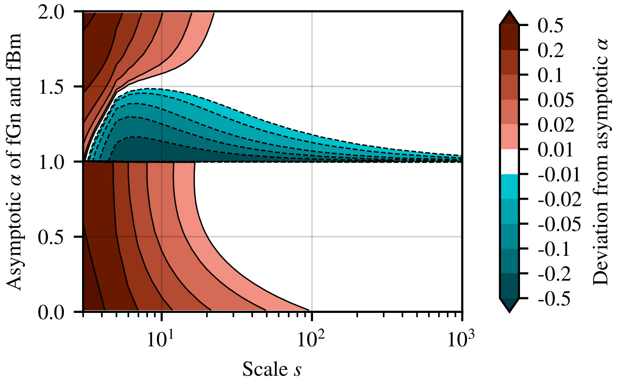

Supplementary Figure S1. Theoretical deviation of the DFA estimate $\alpha(s)$ from asymptotic scaling exponent α for fGn and fBm as a function of the scale s. The deviation is defined as the theoretical scale-dependent exponent $\alpha(s)$ minus the asymptotic scaling exponent α. Note the quasi-logarithmic scale for the deviation.

This matrix $\mathbf{A}$ describes an operator for constructing the squared fluctuations $F_s^2 = \mathbf{X}_{s,w}^\top \mathbf{A} \mathbf{X}_{s,w}$ from the values of the time series $\mathbf{X}_{s,w}$ in window w at the scale s[17].

The autocovariance function for fractional Gaussian noise (fGn) and the variogram for fractional Brownian motion (fBm) with Hurst parameter H are known to be

$$\hat{C}_H^{\text{fGn}}(\tau) = \frac{\sigma^2}{2}\left(|\tau+1|^{2H} - 2|\tau|^{2H} + |\tau-1|^{2H}\right), \tag{13}$$

$$S_H^{\text{fBm}}(\tau) = \sigma^2|\tau|^{2H}, \tag{14}$$

where σ^2 corresponds to the variance of ordinary Gaussian noise[24]. From these correlations and by using Eqs. 8 and 9, the theoretical fluctuation function may be computed for these processes. The spectrum of the scaling exponent $\alpha(s)$ is then obtained from (3). These theoretical results may be utilized for studying the behavior of the DFA method as a function of the scale s. For example, the deviation between the DFA estimate for $\alpha(s)$ and the asymptotic large scale exponent α is visualized in Supplementary Fig. S1 for fGn and fBm. The well-known overestimation of the scaling exponent at the shortest scales is clearly visible, and it is most pronounced in the anticorrelated region with $\alpha < 1/2$. Around the asymptotic value of $\alpha = 1$ there is an abrupt qualitative change as the scaling exponent is suddenly underestimated for an extended range of scales. This has been observed previously[17].

In general, the short scale behavior depends on the details of the underlying process, and hence can be different for other processes such as an autoregressive model AR(p). For more details on the scale dependence of the deviation between asymptotic α and $\alpha(s)$, please see also Ref.[35].

E Numerical Validation of DDFA

Fractional Brownian motion (fBm) and its increments, fractional Gaussian noise (fGn), are commonly utilized for benchmarking DFA (see, e.g., Ref.[36] and references therein). These processes are characterized by the Hurst parameter $0 \le H < 1$, and exhibit long-range correlations with the asymptotic (large-scale) scaling exponents $\alpha = H$ for fGn and $\alpha = H+1$ for fBm[17,24]. Deviations from the asymptotic behavior occur at shorter scales due to the finite length of the samples and the intrinsic bias in DFA due to the detrending. It is, however, possible to compute the exact theoretical scale-dependent scaling exponent $\alpha(s)$ for these processes, as described in Supplementary Appendix D.

We validate the dynamic DFA (DDFA) method by applying it to simulated fGn and fBm, and comparing the results to the theoretically expected values. We utilize the Davies–Harte method, which is an efficient method for simulating these processes with their exact covariance structure[37][3]. We generate 10^3 samples of fGn and fBm of length 10^5 for each value of the Hurst parameter H. From these simulated time series, we compute the dynamic scaling exponent $\alpha(t,s)$ in non-overlapping dynamic segments with various dynamic segment length factors a. The mean difference between the DDFA exponent $\alpha(t,s)$ and the theoretically expected DFA exponent $\alpha(s)$ is illustrated in Supplementary Fig. S2. The general trend is that the limited sample

[3] The accuracy of the simulated time series is established by comparing their joint fluctuation functions (the mean in (2) is taken over the squared fluctuations $F_{s,w}^2$ of all realizations of the simulated time series) to the theoretical fluctuation functions of fGn and fBm.

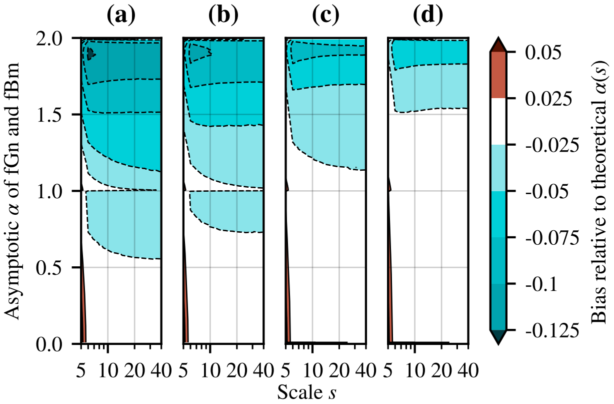

Supplementary Figure S2. Bias of the dynamic detrended fluctuation analysis (DDFA) estimate of $\alpha(t,s)$ relative to its theoretically expected value for fractional Gaussian noise (fGn) and fractional Brownian motion (fBm). Characterized by the Hurst parameter $0 \leq H < 1$, the asymptotic α for these processes is $\alpha = H$ and $\alpha = H + 1$ respectively. Details for computing the theoretical scale-dependent DFA exponent $\alpha(s)$ are provided in Supplementary Appendix D. The bias is defined as the observed $\alpha(t,s)$ minus its theoretically expected value $\alpha(s)$. The dynamic segment length factors a are 4, 5, 7, and 10 in (a-d), respectively.

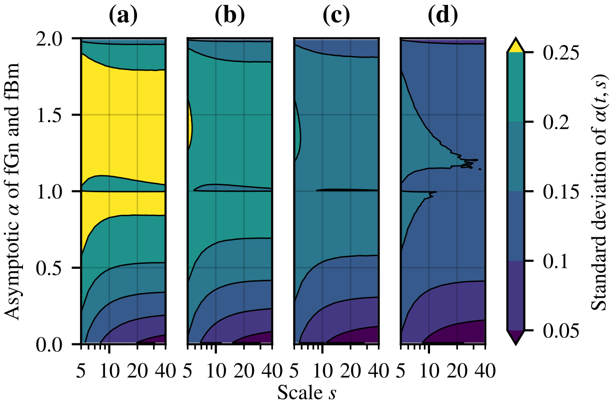

Supplementary Figure S3. Standard deviation of the dynamic detrended fluctuation analysis (DDFA) estimate of $\alpha(t,s)$ for fGn and fBm as a function of the scale s. Characterized by the Hurst parameter $0 \leq H < 1$, the asymptotic α for these processes is $\alpha = H$ and $\alpha = H + 1$ respectively. The dynamic segment length factors a are 4, 5, 7, and 10 in (a-d), respectively.

size results in underestimation of the scaling exponents: the shorter the dynamic segments, the greater the bias. Similarly, processes with larger asymptotic α suffer from larger bias, with a weak discontinuity at $\alpha = 1$ (when the process changes from fGn to fBm). This tendency can be understood as arising from the increased abundance and length of streaks in more correlated time series. This effect is not fully captured by the relatively short segments. However, for the shortest scales (5 and 6), particularly in the anticorrelated region, the exponent is slightly overestimated instead. We also observe that the contour lines for the bias in $\alpha(t,s)$ have nearly converged to a constant value of the asymptotic α already at the scale $s = 40$.

The standard deviation of the DDFA estimation of the exponent α is shown in Supplementary Fig. S3 for segment lengths 4, 5, 7, and 10 in (a-d), respectively. The deviation consistently decreases with the increasing segment length. On the other hand, the deviation reduced as the DFA-1 exponents approach the limits $\alpha = 0$ and $\alpha = 2$, since at these boundaries there is less room for variations. In particular, as α is reduced from $1/2$ towards zero (corresponding to the anticorrelated regime) the deviations are strongly reduced. The local reduction in the deviation just above $\alpha = 1$ is due to the most anticorrelated increments in this regime (see Supplementary Table S3).

Generally, the bias and the standard deviation of the DDFA method are found to be acceptable for our purposes, especially in view of the fact that we have particular interest in the anticorrelated region as underlined below in the results. All our DDFA computations are performed with the dynamic segment length factor $a = 5$. This was found to be a good compromise between

the accuracy of the DDFA method and the dynamical resolution requiring a sufficiently small segment size.

F Additional Heartbeat Correlation Plots

Here we present beat-to-beat (RR) interval (RRI) correlations for all the subjects in the study. In Supplementary Fig. S4 we illustrate the average RRI correlation results as a function of the heart rate aggregated over all the runs for each subject of Group T. The relative heart rate is utilized to better facilitate the comparison between different individuals. Similar correlation plots for the marathons of Group M are shown in Supplementary Fig. S5, along with the correlation landscapes as a function of time during the marathon races. Additionally, we establish the consistency of the anticorrelated bands in the presence of possible trends in Supplementary Fig. S6. We demonstrate this by limiting the analysis to subsets of data where the heart rate within the dynamic segments exhibits subsequently lower and lower standard deviation. The analysis is performed for subject T07, who has the most data.

Each correlation plot consists of pairs of color-coded DDFA (upper panels) and DPACF (lower panels) results. Plots as a function of the heart rate (HR) are based on data that is averaged of dynamic segments whose average HR falls within bins with widths of 0.1 BPM or 0.001 for the absolute and relative HR, respectively. The values for empty bins are linearly interpolated if the gap does not exceed 0.5 BPM (absolute) or 0.005 (relative). The DDFA plots (as a function of the HR) also display the conventional short-scale (4–16 RRIs) scaling exponents α_1 by a semi-transparent black line with error bars (thin bars: standard deviation, thick bars: standard error of the mean, barely visible). The exponent is computed in moving windows of 50 RRIs in HR bins of 2 BPM (absolute) or 0.01 (relative). The DDFA plots displaying the correlation landscapes for single runs show the instantaneous HR with the semi-transparent black line instead, and in the corresponding single run DPACF plots the values that do not pass the non-zero significance test are shown in white.

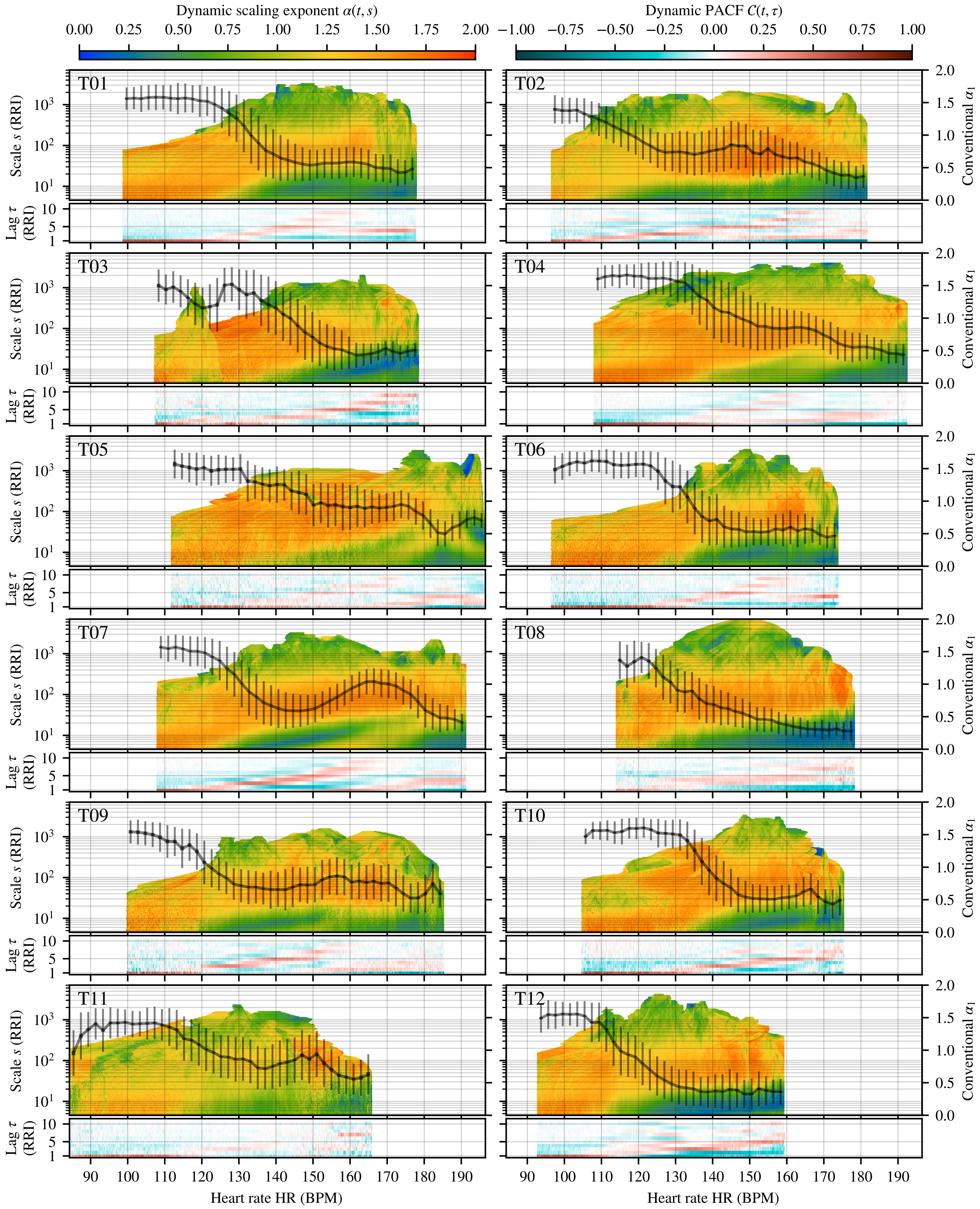

Supplementary Figure S4. Aggregate RRI correlation results for each subject of Group T. For each subject the average DDFA-1 scaling exponents $\alpha(t,s)$ (upper panels) and DPACF-0 correlations $\mathcal{C}(t,\tau)$ (lower panels) as a function of binned relative heart rate. For a detailed explanation about how the data is computed, please see Supplementary Appendix F.

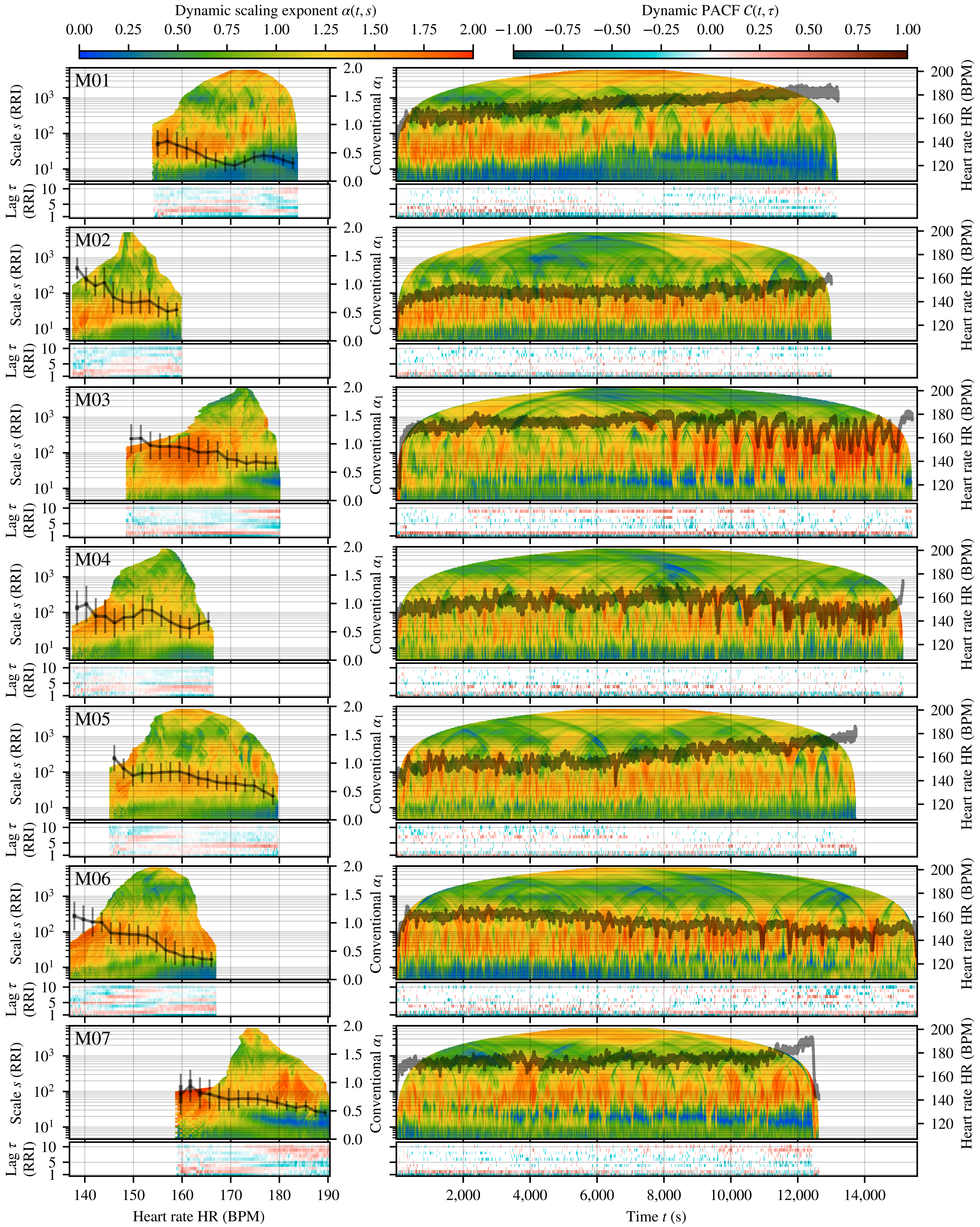

Supplementary Figure S5. Overview of all the marathons in Group M. Left: Average RRI correlation results as a function of binned relative heart rate for each subject of Group M. Right: RRI correlation landscapes of the marathon runs. For each subject the DDFA-1 scaling exponents $\alpha(t,s)$ (upper panels) and DPACF-0 correlations $C(t,\tau)$ (lower panels) are shown. For a detailed explanation about how the data is computed, please see Supplementary Appendix F.

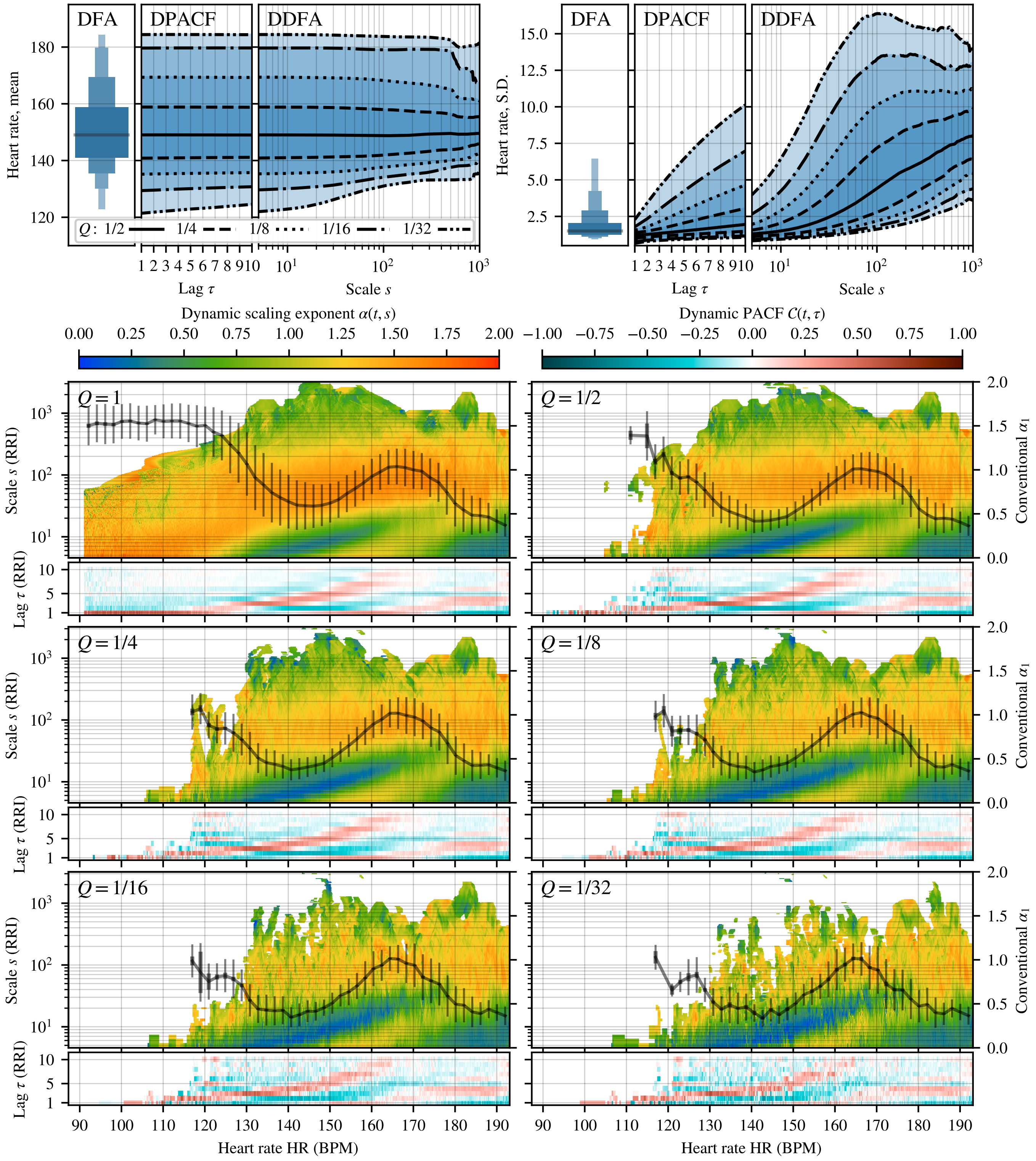

Supplementary Figure S6. Consistency of the results with respect to trends. Top row: different quantiles Q of the mean and standard deviation of the heart rate within the dynamic segments for all the data of subject T07. For DPACF-0 and DDFA-1 the dynamic segment length factor a has values of 10 and 5, respectively, and for conventional DFA the short-range (4–16 beats) scaling exponent is computed in moving windows consisting of 50 RRIs. Lower panels: the average RRI correlation results as a function of the heart rate when the data is limited to dynamic segments with the heart rate standard deviation less than the value for the specified quantiles Q. For a detailed explanation about how the data is computed, please see Supplementary Appendix F.

References

1. Giles, D. A. & Draper, N. Heart rate variability during exercise: A comparison of artefact correction methods. *The J. Strength & Cond. Res.* **32**, DOI: 10.1519/JSC.0000000000001800 (2018).
2. Peng, C.-K. *et al.* Mosaic organization of DNA nucleotides. *Phys. Rev. E* **49**, 1685–1689, DOI: 10.1103/PhysRevE.49.1685 (1994).
3. Santhanam, M. S., Bandyopadhyay, J. N. & Angom, D. Quantum spectrum as a time series: Fluctuation measures. *Phys. Rev. E* **73**, 015201(R), DOI: 10.1103/PhysRevE.73.015201 (2006).
4. Kotimäki, V., Räsänen, E., Hennig, H. & Heller, E. J. Fractal dynamics in chaotic quantum transport. *Phys. Rev. E* **88**, 022913, DOI: 10.1103/PhysRevE.88.022913 (2013).
5. Peng, C., Havlin, S., Stanley, H. E. & Goldberger, A. L. Quantification of scaling exponents and crossover phenomena in nonstationary heartbeat time series. *Chaos: An Interdiscip. J. Nonlinear Sci.* **5**, 82–87, DOI: 10.1063/1.166141 (1995).
6. Goldberger, A. L. *et al.* Fractal dynamics in physiology: Alterations with disease and aging. *Proc. Natl. Acad. Sci.* **99**, 2466–2472, DOI: 10.1073/pnas.012579499 (2002).
7. Kim, J. *et al.* Scaling and correlation properties of RR and QT intervals at the cellular level. *Sci. Reports* **9**, 3651, DOI: 10.1038/s41598-019-40247-9 (2019).
8. Vandewalle, N. & Ausloos, M. Coherent and random sequences in financial fluctuations. *Phys. A: Stat. Mech. its Appl.* **246**, 454 – 459, DOI: 10.1016/S0378-4371(97)00366-X (1997).
9. Hennig, H. *et al.* The nature and perception of fluctuations in human musical rhythms. *PloS one* **6**, e26457, DOI: 10.1371/journal.pone.0026457 (2011).
10. Räsänen, E., Pulkkinen, O., Virtanen, T., Zollner, M. & Hennig, H. Fluctuations of hi-hat timing and dynamics in a virtuoso drum track of a popular music recording. *PLOS ONE* **10**, 1–16, DOI: 10.1371/journal.pone.0127902 (2015).
11. Kantelhardt, J. W., Koscielny-Bunde, E., Rego, H. H., Havlin, S. & Bunde, A. Detecting long-range correlations with detrended fluctuation analysis. *Phys. A: Stat. Mech. its Appl.* **295**, 441–454, DOI: 10.1016/S0378-4371(01)00144-3 (2001).
12. Hu, K., Ivanov, P. C., Chen, Z., Carpena, P. & Stanley, H. E. Effect of trends on detrended fluctuation analysis. *Phys. Rev. E* **64**, 011114, DOI: 10.1103/PhysRevE.64.011114 (2001).
13. Chen, Z., Ivanov, P. C., Hu, K. & Stanley, H. E. Effect of nonstationarities on detrended fluctuation analysis. *Phys. Rev. E* **65**, 041107, DOI: 10.1103/PhysRevE.65.041107 (2002).
14. Heneghan, C. & McDarby, G. Establishing the relation between detrended fluctuation analysis and power spectral density analysis for stochastic processes. *Phys. Rev. E* **62**, 6103–6110, DOI: 10.1103/PhysRevE.62.6103 (2000).
15. Kiyono, K. Establishing a direct connection between detrended fluctuation analysis and fourier analysis. *Phys. Rev. E* **92**, 042925, DOI: 10.1103/PhysRevE.92.042925 (2015).
16. Höll, M. & Kantz, H. The relationship between the detrendend fluctuation analysis and the autocorrelation function of a signal. *The Eur. Phys. J. B* **88**, 327, DOI: 10.1140/epjb/e2015-60721-1 (2015).
17. Løvsletten, O. Consistency of detrended fluctuation analysis. *Phys. Rev. E* **96**, 012141, DOI: 10.1103/PhysRevE.96.012141 (2017).
18. Höll, M., Kantz, H. & Zhou, Y. Detrended fluctuation analysis and the difference between external drifts and intrinsic diffusionlike nonstationarity. *Phys. Rev. E* **94**, 042201, DOI: 10.1103/PhysRevE.94.042201 (2016).
19. Höll, M., Kiyono, K. & Kantz, H. Theoretical foundation of detrending methods for fluctuation analysis such as detrended fluctuation analysis and detrending moving average. *Phys. Rev. E* **99**, 033305, DOI: 10.1103/PhysRevE.99.033305 (2019).
20. Kantelhardt, J. W. *et al.* Multifractal detrended fluctuation analysis of nonstationary time series. *Phys. A: Stat. Mech. its Appl.* **316**, 87–114, DOI: 10.1016/S0378-4371(02)01383-3 (2002).
21. Podobnik, B. & Stanley, H. E. Detrended cross-correlation analysis: A new method for analyzing two nonstationary time series. *Phys. Rev. Lett.* **100**, 084102, DOI: 10.1103/PhysRevLett.100.084102 (2008).
22. Kiyono, K. & Tsujimoto, Y. Nonlinear filtering properties of detrended fluctuation analysis. *Phys. A: Stat. Mech. its Appl.* **462**, 807 – 815, DOI: 10.1016/j.physa.2016.06.129 (2016).
23. Hurst, H. E. The problem of long-term storage in reservoirs. *Int. Assoc. Sci. Hydrol. Bull.* **1**, 13–27, DOI: 10.1080/02626665609493644 (1956).

24. Mandelbrot, B. & Van Ness, J. Fractional brownian motions, fractional noises and applications. *SIAM Rev.* **10**, 422–437, DOI: 10.1137/1010093 (1968).

25. Ge, E. & Leung, Y. Detection of crossover time scales in multifractal detrended fluctuation analysis. *J. Geogr. Syst.* **15**, 115–147, DOI: 10.1007/s10109-012-0169-9 (2013).

26. Habib, A. *et al.* Temporal scaling phenomena in groundwater-floodplain systems using robust detrended fluctuation analysis. *J. Hydrol.* **549**, 715 – 730, DOI: 10.1016/j.jhydrol.2017.04.034 (2017).

27. Viswanathan, G. M., Peng, C.-K., Stanley, H. E. & Goldberger, A. L. Deviations from uniform power law scaling in nonstationary time series. *Phys. Rev. E* **55**, 845–849, DOI: 10.1103/PhysRevE.55.845 (1997).

28. Echeverría, J. C. *et al.* Interpretation of heart rate variability via detrended fluctuation analysis and $\alpha\beta$ filter. *Chaos: An Interdiscip. J. Nonlinear Sci.* **13**, 467–475, DOI: 10.1063/1.1562051 (2003).

29. Castiglioni, P., Parati, G., Civijian, A., Quintin, L. & Rienzo, M. D. Local scale exponents of blood pressure and heart rate variability by detrended fluctuation analysis: Effects of posture, exercise, and aging. *IEEE Transactions on Biomed. Eng.* **56**, 675–684, DOI: 10.1109/TBME.2008.2005949 (2009).

30. Xia, J., Shang, P. & Wang, J. Estimation of local scale exponents for heartbeat time series based on DFA. *Nonlinear Dyn.* **74**, 1183–1190, DOI: 10.1007/s11071-013-1033-2 (2013).

31. Molkkari, M. & Räsänen, E. Robust estimation of the scaling exponent in detrended fluctuation analysis of beat rate variability. In *Computing in Cardiology*, DOI: 10.22489/CinC.2018.219 (2018).

32. Box, G. E., Jenkins, G. M. & Reinsel, G. C. *Time Series Analysis, Forecasting and Control*. Wiley Series in Probability and Statistics (Wiley, 2008), 4 edn.

33. Meyer, P. G. & Kantz, H. Inferring characteristic timescales from the effect of autoregressive dynamics on detrended fluctuation analysis. *New J. Phys.* **21**, 033022, DOI: 10.1088/1367-2630/ab0a8a (2019).

34. Bryce, R. M. & Sprague, K. B. Revisiting detrended fluctuation analysis. *Sci. reports* **2**, 315, DOI: 10.1038/srep00315 (2012).

35. Molkkari, M. *Advanced Methods in Detrended Fluctuation Analysis with Applications in Computational Cardiology*. Master's thesis, Tampere University (2019).

36. Shao, Y.-H., Gu, G.-F., Jiang, Z.-Q., Zhou, W.-X. & Sornette, D. Comparing the performance of FA, DFA and DMA using different synthetic long-range correlated time series. *Sci. Reports* **2**, 835, DOI: 10.1038/srep00835 (2012).

37. Davies, R. B. & Harte, D. S. Tests for Hurst effect. *Biometrika* **74**, 95–101, DOI: 10.1093/biomet/74.1.95 (1987).
